# Heart rate as an early predictor of severe cardiomyopathy and increased mortality in peripartum cardiomyopathy

**DOI:** 10.1002/clc.23782

**Published:** 2022-02-07

**Authors:** Ryan Cooney, John R. Scott, Madeline Mahowald, Elizabeth Langen, Garima Sharma, David P. Kao, Melinda B. Davis

**Affiliations:** ^1^ Department of Internal Medicine University of Michigan Ann Arbor Michigan USA; ^2^ Department of Anesthesiology University of Michigan Ann Arbor Michigan USA; ^3^ Minneapolis Heart Institute Abbott Northwestern Hospital Minneapolis Minnesota USA; ^4^ Department of Obstetrics and Gynecology University of Michigan Ann Arbor Michigan USA; ^5^ Department of Internal Medicine Hopkins Ciccarone Center for Prevention of Cardiovascular Disease, Division of Cardiology Baltimore Maryland USA; ^6^ Department of Internal Medicine, Division of Cardiology University of Colorado School of Medicine Aurora Colorado USA; ^7^ Department of Internal Medicine, Division of Cardiovascular Medicine University of Michigan Ann Arbor Michigan USA

**Keywords:** heart failure, nonischemic cardiomyopathy, outcomes, peripartum cardiomyopathy, pregnancy, risk factors

## Abstract

**Background:**

Delays in diagnosis of peripartum cardiomyopathy (PPCM) are common and are associated with worse outcomes; however, few studies have addressed methods for improving early detection.

**Hypothesis:**

We hypothesized that easily accessible data (heart rate [HR] and electrocardiograms [ECGs]) could identify women with more severe PPCM and at increased risk of adverse outcomes.

**Methods:**

Clinical data, including HR and ECG, from patients diagnosed with PPCM between January 1998 and July 2016 at our institution were collected and analyzed. Linear and logistic regression were used to analyze the relationship between HR at diagnosis and the left ventricular ejection fraction (LVEF) at diagnosis. Outcomes included overall mortality, recovery status, and major adverse cardiac events.

**Results:**

Among 82 patients meeting inclusion criteria, the overall mean LVEF at diagnosis was 26 ± 11.1%. Sinus tachycardia (HR > 100) was present in a total of 50 patients (60.9%) at the time of diagnosis. In linear regression, HR significantly predicted lower LVEF (*F* = 30.00, *p* < .0001). With age‐adjusted logistic regression, elevated HR at diagnosis was associated with a fivefold higher risk of overall mortality when initial HR was >110 beats per minute (adjusted odds ratio 5.35, confidence interval 1.23–23.28), *p* = .025).

**Conclusion:**

In this study, sinus tachycardia in women with PPCM was associated with lower LVEF at the time of diagnosis. Tachycardia in the peripartum period should raise concern for cardiomyopathy and may be an early indicator of adverse prognosis.

**Abbreviations**
ACE: angiotensin‐converting enzyme
BNP: brain natriuretic peptide
ECG: electrocardiogram
HR: heart rate
LVAD: left ventricular assist device
LVEF: left ventricular ejection fraction
MACE: major adverse cardiac events
PPCM: peripartum cardiomyopathy

## 1 INTRODUCTION

Pregnancy‐related mortality remains a leading cause of death among women across the world.^1^ Nearly two‐thirds of pregnancy‐related deaths occur around the time of delivery or within the first 6 weeks postpartum,^2^ and as many as two‐thirds of these deaths are considered to be preventable.^3^, ^4^, ^5^ Cardiovascular disease is the leading cause of pregnancy‐related deaths in the United States.^6^, ^7^ Between 2014 and 2017, cardiovascular conditions comprised 15.5% of maternal deaths in the United States.^6^, ^7^ Cardiomyopathy was responsible for 11.5% of deaths^7^ and is currently the leading cause of death between 6 weeks and 1 year following delivery.^8^

Peripartum cardiomyopathy (PPCM) is an idiopathic form of left ventricular systolic dysfunction that causes clinical heart failure in women during the last trimester of pregnancy or the early postpartum period.^9^, ^10^ Although most patients recover, mortality averages 5%–15% in the United States^9^, ^10^, ^11^ and can be even higher in other areas of the world.^10^, ^12^ Survivors may suffer significant morbidity related to persistently decreased cardiac function, including the need for multiple medications, frequent hospital readmissions, and poor quality of life.^13^ Numerous studies have shown that left ventricular ejection fraction (LVEF) less than 30% at the time of diagnosis portends a worse prognosis, with lower rates of recovery and increased risk of adverse events.^9^, ^11^

Delays in diagnosis are associated with lower LVEF, higher rates of adverse outcomes, and lower rates of recovery.^14^, ^15^ Early detection of PPCM is challenging because symptoms of heart failure mimic those of normal pregnancy, especially late in the third trimester and early after delivery.^9^, ^10^ Despite the clear association between late diagnosis and worse outcomes, few studies have addressed methods for improving early detection. One risk prediction tool was recently validated but includes several demographic and comorbidity variables that would be most effective if integrated into an electronic medical record.^16^ We, therefore, sought to determine whether simple bedside clinical data (heart rate [HR]) and electrocardiogram [ECG]) could effectively identify women with more severe forms of PPCM and increased risk for adverse outcomes. We utilized a well‐defined clinical cohort of women with PPCM to test our hypothesis that significant elevations in HR or abnormal ECG findings in peripartum women would be indicators of more severe cardiomyopathy and, consequently, increased risk of adverse outcomes.

## 2 METHODS

### 2.1 Study population

**Figure 1:**
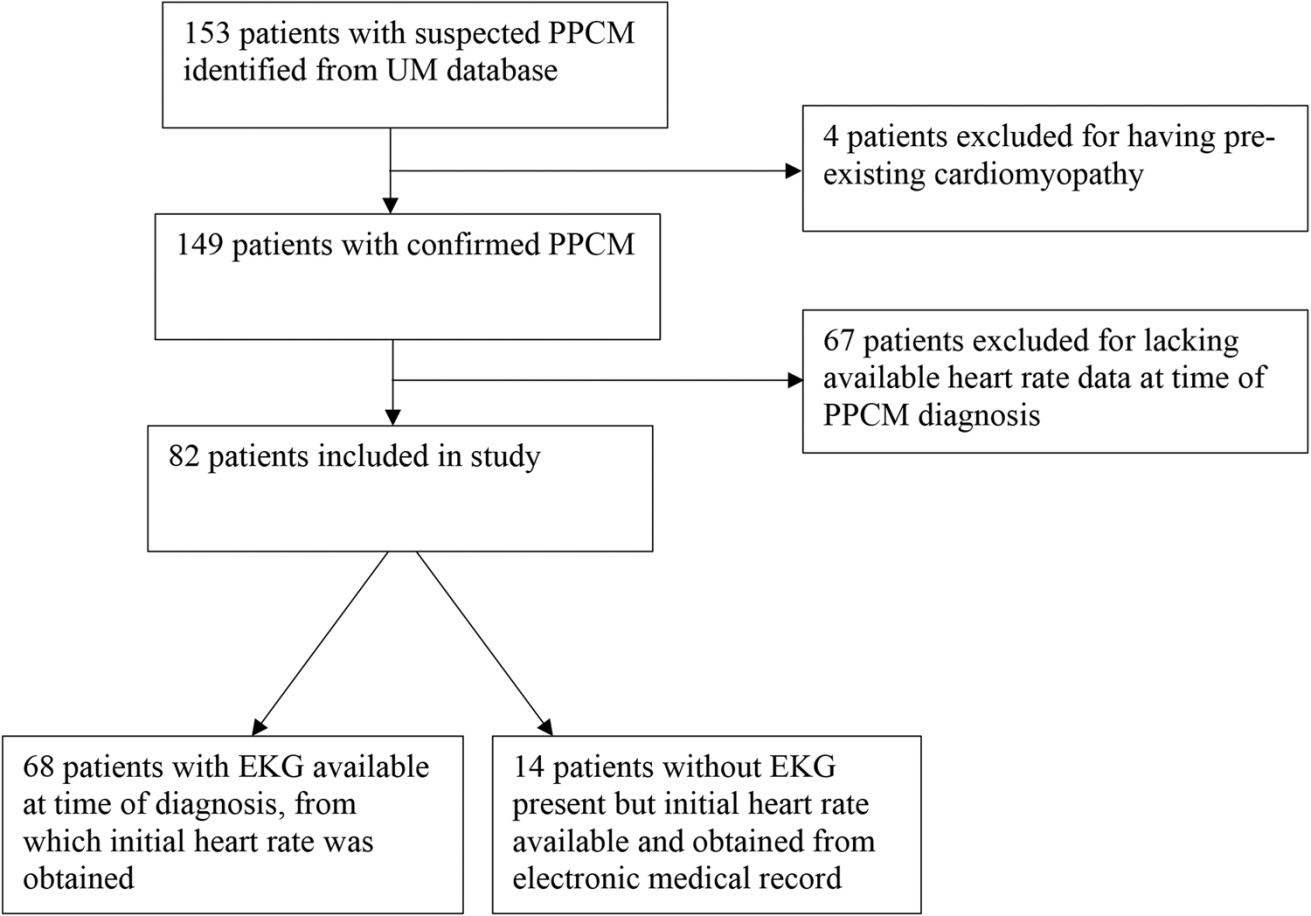
Flowchart of study sample inclusion and exclusion criteria. This flowchart demonstrates the inclusion and exclusion criteria used to determine the final sample size. EKG, electrocardiogram; PPCM, peripartum cardiomyopathy

Approval for the study and waiver of written informed consent was obtained from the University of Michigan Institutional Review Board. Patients diagnosed with PPCM (January 1, 1998–July 31, 2016) were identified using the University of Michigan Electronic Medical Record Search Engine (EMERSE). Details of the search engine function and performance have been previously described elsewhere.^17^ EMERSE was used to search for all patient encounters with the following terms: pregnancy‐associated cardiomyopathy, post‐partum cardiomyopathy, postpartum cardiomyopathy, peri‐partum cardiomyopathy, peripartum cardiomyopathy, and PPCM. Each chart was manually reviewed in detail. ECG and HR data were extracted by a physician reviewer initially blinded to associated outcomes and LVEF. Patients were included if they met the diagnostic criteria for PPCM defined according to the currently accepted definition as an idiopathic cardiomyopathy with initial LVEF < 45% presenting towards the end of pregnancy or in the months following delivery.^9^, ^10^, ^18^ Only patients diagnosed at our institution with sufficient imaging data to confirm the diagnosis and with a minimum of 6 months of follow‐up were included (Figure 1). Exclusion criteria included prior chemotherapy, chest radiation, congenital heart disease, valvular disease, family history suggestive of hereditary dilated cardiomyopathy, preceding febrile illnesses, and recent or recurrent illicit drug use. Race was self‐identified in the medical record.

### 2.2 Data collection

Demographic information, HR, LVEF, and outcomes data were collected for all patients. HR at the time of diagnosis (baseline HR) was determined by ECG on presentation if it was available, or by initial HR obtained on examination and documented in the electronic medical record at the visit during which PPCM was diagnosed. Sinus tachycardia was defined as HR ≥ 100 beats per minute (bpm); this was further specified to identify patients with HR >110 and >120 bpm. LVEF measurements at the time of diagnosis and at 6 and 12 months follow‐up were obtained. Recovery of systolic function was defined as final LVEF ≥ 55%.

For the subset of patients who had ECGs available (*n* = 68), conventional parameters including ventricular rate, QRS axis, PR interval, QRS duration, QTc interval, and T‐wave amplitude in lead aVR were calculated. ECGs were also analyzed for the presence of arrhythmia, pathologic Q waves (defined as a Q wave >2 mm deep or >40 ms wide), left or right atrial enlargement, right or left bundle branch block, T‐wave inversion, left ventricular hypertrophy, or poor R‐wave progression (defined as R < S in V4). QTc interval was considered prolonged if it was >460 ms. A positive T wave in lead aVR was defined as a wave with a positive deflection >0 mV. An ECG was considered abnormal if any interval was prolonged or if any of the above findings were present. Both sinus tachycardia (ventricular rate >100 bpm) and sinus bradycardia (ventricular rate < 60 bpm) were considered abnormal findings. All ECG findings were confirmed by a board‐certified cardiologist and also reviewed by a physician investigator blinded to the clinical outcomes.

### 2.3 Patient characteristics and outcomes

Data on patient demographics (age, race), pregnancy (gravida, parity, mode of delivery), and comorbidities (hypertensive disorders of pregnancy) were extracted and compared between patients with HR >100, 110, and 120 bpm. Baseline characteristics included LVEF at the time of diagnosis. Outcomes included overall mortality, recovery status (LVEF ≥ 55%), and major adverse cardiac events (MACEs) defined as a composite of left ventricular assist device (LVAD) placement, transplant, or death.

### 2.4 Statistical analysis

Categorical variables were compared using the Pearson *χ* ^2^ test and reported as frequency (*n*) ± percentage (%). Linear regression was used to assess the associations between continuous variables. Continuous outcome results are reported as mean ± SD or SE. Odds ratios (OR) for dichotomous outcomes were calculated using binary logistic regression and reported as 95% confidence interval (CI). In addition, logistic regression was performed to control for patient age and is presented as an adjusted odds ratio (AOR). All *p* values are two‐sided, with *p* < .05 considered significant. Statistical analyses were performed using SPSS 27 (IBM Corp).

## 3 RESULTS

### 3.1 Demographic data and clinical characteristics

Baseline demographic data and clinical characteristics are shown in Table 1. The mean age of the study population at diagnosis was 29.9 ± 7.3 years and most patients had a gravidity of 1 (39.0%) or 2 (28.0%). The majority of patients were White (67.5%), with the remainder being African American (25.0%), Asian (4.9%), or of mixed ancestry (4.9%). In 41.5% of patients, pregnancy was complicated by hypertension (gestational or chronic) or pre‐eclampsia. Following the diagnosis, the majority of patients received treatment with beta‐blocker (89.0%), loop diuretic (97.6%), or angiotensin‐converting enzyme (ACE) inhibitor/angiotensin receptor blocker (92.7%) medications. Patients presenting with a HR > 120 bpm at diagnosis were more likely to subsequently require inotropes (*n* = 7, 38.9%, OR = 3.75, 95% CI 1.15–12.22, *p* = .028) and digoxin (*n* = 14, 70.0%, OR = 4.56, 95% CI 1.53–13.57, *p* = .006). No significant associations were observed between elevated HR and patient demographic parameters (Table 1).

**Table 1:** Demographic data for all PPCM patients: overall cohort and by HR at diagnosis

	Total	HR > 100	*p* value	HR > 11	*p* value	HR > 120	*p* value
*n* (%)	82 (100)	50 (60.9)		35 (42.7)		20 (24.4)	
Age at diagnosis, mean (SD)	29.9 (7.3)	29.8 (7.4)	.818	30.2 (7.6)	.768	28.5 (7.1)	.964
Diagnosed postpartum	75 (91.5)	45 (60.0)	.557	31 (41.3)	.0425	18 (24)	.788
*Race*			.509		.244		.795
White	54 (65.0)	31 (64.6)	.700	25 (75.8)	.453	14 (73.7)	.547
Black	20 (25.0)	12 (25.0)	.841	5 (15.)2	.104	4 (21.1)	.599
Other	6 (7.5)	5 (10.5)	.246	3 (9.1)	.863	1 (5.3)	.623
*Gravida*			.994		.894		.999
1	32 (39.0)	20 (40.0)		13 (37.1)		9 (45.0)	
2	23 (28.0)	14 (28.0)		11 (31.4)		7 (35.0)	
3	8 (9.8)	5 (10.0)		5 (14.3)		3 (15.0)	
4	9 (11.0)	5 (10.0)		3 (8.6)		0	
5	3 (3.7)	2 (4.0)		1 (2.9)		0	
6	5 (6.1)	2 (4.0)		1 (2.9)		1 (5.0)	
7	1 (1.2)	1 (2.0)		1 (2.9)		0	
8	1 (1.2)	1 (2.0)		0		0	
*Para*			.934		.897		.403
0	4 (4.9)	3 (6.0)		3 (8.6)		3 (15.0)	
1	35 (42.7)	20 (40.0)		13 (37.1)		7 (35.0)	
2	24 (29.3)	14 (28.0)		11 (31.4)		8 (40.0)	
3	11 (13.4)	6 (12.0)		5 (14.3)		1 (5.0)	
4	6 (7.3)	5 (10.0)		2 (5.7)		0	
5	0	0		0		0	
6	1 (1.2)	1 (1.2)		1 (2.9)		1 (5.0)	
7	1 (1.2)	1 (1.2)		0		0	
*Delivery*							
Vaginal	45 (42.7)	16 (32.0)	.021	14 (41.2)	.690	10 (52.6)	.371
Cesarean	35 (54.9)	32 (64.0)	.023	20 (58.5)	.690	9 (47.4)	.373
Pre‐eclampsia	22 (26.8)	16 (32.0)	.191	12 (34.3)	.192	4 (20.0)	.431
Hypertensive disorders of pregnancy	34 (41.5)	25 (50.0)	.053	18 (51.4)	.116	7 (35.0)	.501
Beta‐blocker	73 (89.0)	44 (88.0)	.711	.711	.711	16 (80.0)	.150
ACEi/ARB	76 (92.7)	44 (88.0)	1.000	30 (85.7)	.069	17 (85.0)	.149
Loop diuretic	80 (97.6)	49 (98.0)	.749	.749	.833	19 (95.0)	.417
Spironolactone	32 (39.0)	24 (48.0)	.040	16 (45.7)	.285	10 (50.0)	.250
Hydralazine	12 (14.6)	8 (16.0)	.663	8 (22.9)	.079	4 (20.0)	.438
Nitrates	11 (13.4)	6 (12.0)	.639	6 (17.1)	.397	2 (10.0)	.608
Digoxin	35 (42.7)	25 (50.0)	.097	20 (57.1)	.024	14 (70.0)	.006
Inotropes	16 (19.5)	13 (27.1)	.063	11 (33.3)	.017	7 (38.9)	.028
Bromocriptine	2 (2.4)	2 (4.0)	1.000	2 (5.7)	1.000	1 (5.0)	.417
IABP	4 (4.9)	4 (8.2)	1.0	4 (11.8)	1.000	4 (21.1)	1.000

*Note*: χ^2^ test was performed; the exact test was performed for variables with groups *n* < 5. Age at diagnosis is reported as age (mean ± SD). All other data are reported as *n* (%), unless otherwise indicated. Hypertensive disorders of pregnancy include pre‐existing hypertension, gestational hypertension, and/or pre‐eclampsia.

Abbreviations: ACEi, angiotensin‐converting enzyme inhibitor; ARB, angiotensin receptor blocker; HR, heart rate; IABP, intra‐aortic balloon pump; PPCM, peripartum cardiomyopathy.

### 3.2 Overall outcomes data

Overall, 75.6% of patients had full recovery of left ventricular function (LVEF ≥ 55%), with 64.4% of those recovered by 6 months and 81.4% recovered by 12 months. During the overall follow‐up, a total of 17 (20.7%) patients ultimately had at least one MACE; 14 out of these 17 patients (82.3%) had LVEF < 30% at diagnosis. Of all the patients with MACE, six patients required LVAD and two patients underwent cardiac transplantation; one additional patient, not included in MACE, was listed for transplant but had not received this at the time of last follow‐up. Death accounted for the majority of MACE, with a total of 11 patient deaths in the study population for an overall mortality of 13.4% over the follow‐up period.

### 3.3 Comparison of ejection fraction with outcomes

At diagnosis, 40 (49%) and 60 (73%) patients had a LVEF <25% and <35%, respectively. The overall sample mean (±SD) LVEF was 26 ± 11.1%. A total of 59 (72%) patients recovered, with 38 (63.3%) of those recovered having an initial LVEF of <35% and 23 (57.5%) having a LVEF of <25%. Of the 28% of patients who did not fully recover, nearly all (95%) had a LVEF < 35% at the time of diagnosis, and nearly three‐quarters (73.7%) had a LVEF < 25%. In logistic regression analysis, LVEF < 35% at diagnosis was associated with lower odds of recovery; patients having LVEF <25% and <35% were found to be 77% (AOR = 0.23, 95% CI 0.07–0.72, *p* = .012) and 90% (AOR = 0.10 95% CI 0.01–0.81, *p* = .031) less likely to recover, respectively, than patients with higher LVEF. Patients with LVEF < 25% at diagnosis were also seven times more likely to have a MACE (AOR = 6.97, 95% CI 1.79–27.16, *p* = .005).

### 3.4 Comparison of HR with LVEF at diagnosis and 6 months follow‐up

**Figure 2:**
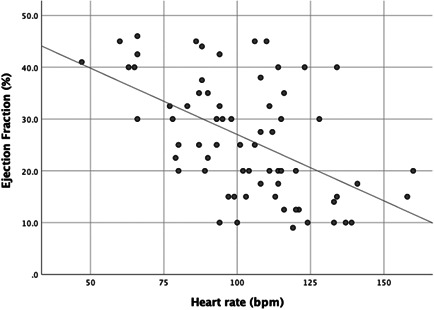
Relationship between ejection fraction and heart rate at diagnosis. This figure shows the relationship between HR (independent variable) and LVEF (dependent variable). Using linear regression, higher HR was associated with lower LVEF. HR, heart rate; LVEF, left ventricular ejection fraction

At the time of diagnosis, sinus tachycardia was present in 50 patients (61%), with a mean sample HR (±SD) of 106 ± 24 bpm. Sinus tachycardia (HR > 100 bpm) had a significant association with lower LVEF at diagnosis (Table 2). With linear regression, higher HR predicted lower LVEF (*F* = 30.00, *p* < .0001) where for every 1 bpm increase in HR, LVEF percentage decreased 0.24 ± 0.04 units (Figure 2). In age‐adjusted logistic regression, patients with sinus tachycardia were five times more likely to have LVEF < 25% at diagnosis (AOR = 5.24, 95% CI 1.93–14.24, *p* = .001); this was seven times more likely if HR was >110 bpm (OR = 6.81, 95% CI 2.55–18.19, *p* = .000).

**Table 2:** Association of elevated HR with decreased LVEF

	EF ≥ 35% at diagnosis (*n* = 22)	EF < 35% at diagnosis (*n* = 60)	OR (95% CI)	*p* value
HR < 100 (*n* = 32)	13 (59)	19 (32)	0.321 (0.12–0.88)	.027
HR ≥ 100 (*n* = 50)	9 (41)	41 (68)	3.12 (1.14–8.55)	.027
HR > 110 (*n* = 35)	5 (22.7)	31 (51.7)	4.81 (1.46–15.91)	.010
HR > 120 (*n* = 20)	2 (9)	18 (30)	4.29 (0.91–20.29)	.067

*Note*: Values are *n* (%), unless otherwise indicated.

Abbreviations: CI, confidence interval; EF, ejection fraction; HR, heart rate; LVEF, left ventricular ejection fraction; OR, odds ratio.

### 3.5 Comparison of HR with LVEF recovery, MACE, and mortality

In the analysis of outcomes, sinus tachycardia at diagnosis was significantly associated with recovery at 6 months (Table 3), such that patients with HR of 100 bpm or greater were 63% less likely to recover by this time (OR = 0.37, 95% CI 0.14–0.99, *p* = .049). However, this association was not statistically significant after 1 year (*p* = .064). HR at diagnosis was not associated with the composite MACE outcome (LVAD, transplantation, and death). Univariable logistic regression of initial HR and overall mortality revealed that sinus tachycardia with HR elevated >110–120 bpm was associated with increased mortality. The odds of death were four times higher when initial HR was >120 bpm (OR 4.17, 95% CI 1.06–16.35, *p* = .041) and five times higher when initial HR was >110 bpm (OR = 4.95, 95% CI 1.19–20.67, *p* = .028).

**Table 3:** Association between elevated HR and recovery

	Recovered at 6 months (*n* = 38)	*p* value	Recovered at 12 months (*n* = 48)	*p* value	Recovered eventually (*n* = 59)	*p* value
HR < 100 (*N* = 32)	18 (47.4)	.049	22 (45.8)	.073	27 (46)	.063
HR ≥ 100 (*N* = 50)	20 (52.6)	.049	26 (54.2)	.073	32 (54)	.063
HR > 110 (*N* = 35)	15 (39)	.363	19 (40)	.491	22 (37)	.240
HR > 120 (*N* = 20)	9 (24)	.687	12 (25)	.925	12 (20)	.316

*Note*: Values are *n* (%), unless otherwise indicated.

Abbreviations: bpm, beats per minute; HR, heart rate.

### 3.6 ECG characteristics and subgroup analysis

Within the study population, there were 68 patients with complete ECG data available from the time of diagnosis. The majority of ECGs (82%) had at least one abnormal finding (Table S1) with the most prevalent being sinus tachycardia, which was present in 66% of the abnormal ECGs. The overall mean (±SD) HR for abnormal ECGs was 108 ± 21.9 bpm. No patients had atrial fibrillation. The other most common abnormal findings included prolonged QTc (48% of abnormal ECGs), with an average QTc of 455 ± 41.99 ms, T‐wave inversion (30%), and pathologic Q waves (28.5%). Ten ECGs (18%) met the criteria for having a positive T wave in lead aVR. There were nine patients with a left atrial enlargement (18% of abnormal ECGs) and five with left ventricular hypertrophy (9%). Only one patient had a left bundle branch block and there were no ECGs with right bundle branch block present. There were no significant differences in demographic factors among women with normal vs abnormal ECGs.

## 4 DISCUSSION

This study demonstrates that tachycardia in the peripartum period is a simple, early, independent predictor of severe cardiac dysfunction among women with PPCM. In this well‐defined cohort, sinus tachycardia at the time of diagnosis was associated with a lower initial LVEF, a lower likelihood of recovery, and a significantly increased risk of overall mortality, especially if the HR was >110 bpm (Central Illustration). While other ECG findings such as prolonged QTc, T‐wave inversion, and pathologic Q waves were common in our PPCM cohort, only sinus tachycardia was significantly associated with outcomes. Although tachycardia is common among women at the end of pregnancy or early after delivery, we found that among women diagnosed with PPCM, sinus tachycardia (HR > 110 bpm) was associated with a fivefold increased risk of mortality.

Sinus tachycardia is common during and after pregnancy, but significant elevations in HR are uncommon. Green and colleagues documented postpartum vital signs for 909 healthy pregnant women from their second trimester through 2 weeks postpartum to determine normal postpartum vital sign ranges.^19^ The HR was highest on the day of birth with a median HR of 84 bpm (50th percentile) and a maximum HR of 110 bpm (97th percentile). The average HR decreases after delivery and at 2 weeks postpartum; the median HR was 75 bpm. The 97th percentile was HR of 105 bpm. Since most women are diagnosed in the postpartum time frame, this further supports our findings that a resting HR > 110 bpm may warrant additional attention, especially if there are any accompanying symptoms of heart failure.

Similar to the sepsis initiative of recent years, there has been a focus within obstetrics on identifying early warning signs that may help differentiate these healthy women from those with critical illness in the peripartum period to decrease maternal mortality related to delays in diagnosis and treatment. Mhyre and colleagues proposed the Maternal Early Warning Criteria, a set of vital sign parameters and clinical signs/symptoms that should prompt physician evaluation of a pregnant patient deemed to be at risk of a critical illness such as hemorrhage, sepsis, hypertensive crisis, or heart failure.^20^ One of the criteria included is HR > 120 bpm, which the authors argue should raise concern for the possibility of a life‐threatening cardiac problem among other diagnoses. Our finding that sinus tachycardia (especially HR > 110–120 bpm) predict severe cardiomyopathy and increased mortality in PPCM further supports the recommendation that elevated HR should draw attention in a pregnant or postpartum woman.

Our study also supports prior findings that lower LVEF at the time of diagnosis is strongly associated with a worse prognosis.^11^, ^21^, ^22^, ^23^ One of the most prominent studies in this regard is the Investigations of Pregnancy‐Associated Cardiomyopathy study, which demonstrated that an initial LVEF < 30% was significantly associated with a persistently low LVEF and lack of recovery at 12‐month follow‐up.^21^ Similarly, we found that women with an initial LVEF < 25% were 75% less likely to fully recover. In a study by Goland et al.,^22^ LVEF < 25% significantly predicted an increase in major adverse events by fivefold; in our study, patients were seven times more likely to have MACE with this initial LVEF.

A few prior studies have investigated the prognostic value of the ECG as a tool to identify PPCM patients at increased risk for poor outcomes.^24^, ^25^, ^26^, ^27^ Several ECG abnormalities have been identified in PPCM,^27^, ^28^ but the results have been variable as to the significance of specific ECG findings. In a study by Honigberg et al.,^26^ the presence of left atrial enlargement on baseline ECG portended a lower LVEF at both 6‐ and 12‐month follow‐up; Ekizler et al.^24^ found that a positive T wave in lead aVR was a strong predictor of adverse cardiac outcomes. A recent study by Hoevelmann et al.^25^ found that prolonged QTc at presentation predicted poor 6‐month outcomes, while sinus tachycardia predicted poor outcomes at 12 months. Although our study investigated the relevance of each of these ECG findings, only sinus tachycardia was found to be significantly associated with lower initial LVEF and increased mortality. Overall, our findings and those of others show that various ECG abnormalities are common in women with PPCM; however, a normal ECG is not sufficiently sensitive to rule out the diagnosis.

While the above study by Hoevelmann et al.^25^ and another recent study by Mbakwem et al.^29^ also investigated the relationship between tachycardia and outcomes, both designated sinus tachycardia as HR of 100 bpm or greater and used a cutoff of LVEF < 35% to evaluate systolic function. This is in contrast to our study in which we further evaluated HR >110 and >120 bpm to characterize a linear relationship between increasing degrees of tachycardia and increased severity of cardiomyopathy (Figure 2). Another prior study, from a registry in Nigeria, reported that sinus tachycardia was associated with increased mortality; however, most patients in this study were diagnosed late at 3–6 months postpartum, likely representing those who had failed to recover and therefore already at higher risk of death.^30^

Because such delays in diagnosis are common and are associated with worse outcomes,^14^, ^15^ simple and easily accessible tools for assessing increased risk in PPCM would be very useful. Most previously published prediction tools for PPCM would likely require integration within electronic health systems to be utilized.^16^ A simpler risk score was investigated by Karaye et al.,^31^ who found that having at least two of three specified ECG disturbances (HR > 100 bpm, presence of ST–T‐wave abnormalities, QRS duration ≥ 110 ms) helped predict PPCM diagnosis in Nigerian women presenting with heart failure symptoms. The authors in this study highlight the usefulness of such a tool, especially in resource‐poor settings. Our study suggests that assessing women with increased HR could be a simple and effective way to identify women at increased risk for severe cardiomyopathy earlier in their course.

Our study has both strengths and weaknesses. We studied a cohort of well‐characterized women with confirmed diagnoses of PPCM and highly detailed information about HR, ECG findings, LVEF, and outcomes. We employed strict exclusion criteria to analyze patients with sufficient information and accurate diagnoses. Limitations include the variable nature of vital signs and that changes in HR over time were not available. The retrospective nature of this study also limited the availability of additional patient clinical, laboratory, and echocardiographic data that may have further described the baseline characteristics of this cohort. It is worth noting that this cohort may represent a particularly high‐risk patient population as evidenced by the high proportion of patients with baseline LVEF < 25% (49%), mortality rate of 13.4%, six patients requiring LVAD, and two patients undergoing heart transplantation. Additionally, our study focused on the use of EKG and HR, but future studies could assess the implication of other clinical variables that could also predict adverse outcomes.

In conclusion, we suggest that elevated HR in a pregnant woman, especially above 110 bpm, should prompt health professionals to assess for signs and symptoms of heart failure. When there is clinical concern, additional testing such as BNP and echocardiogram are required to confirm the diagnosis. Future investigation is needed to determine whether tachycardia would reliably identify women with heart failure in a general population of peripartum women, potentially leading to reductions in maternal morbidity and mortality.

### 4.1 Competencies in medical knowledge

Sinus tachycardia is common during pregnancy and after delivery; however, elevated HR may be an early predictor of severe cardiomyopathy and early mortality. Elevated HR in a pregnant woman, especially above 110 bpm, should prompt health professionals to assess for signs and symptoms of heart failure and perform additional testing such as BNP and echocardiogram when warranted.

### 4.2 Translational outlook

As maternal mortality from the cardiovascular disease continues to rise, screening tools for the early identification of women at increased risk are needed. Future investigation is needed to determine whether tachycardia could reliably identify women with heart failure in a general population of peripartum women, and lead to reductions in morbidity and mortality.

## CONFLICT OF INTERESTS

The authors declare that there are no conflict of interests.

## Supporting information

Supporting information.

## Data Availability

The data that support the findings of this study are available from the corresponding author (M. B. D.) upon reasonable request.
