# Supplementary material for: Heart rate as an early predictor of severe cardiomyopathy and increased mortality in peripartum cardiomyopathy
Source: Clin Cardiol. 2022 Feb 7;45(2):205–13. doi: 10.1002/clc.23782 (PMC8860487; doi:10.1002/clc.23782)
Supplement: Supplementary file 1 — Supporting information. [file CLC-45-205-s001.docx]

**SUPPLEMENTARY TABLE 1: ECG FINDINGS IN WOMEN WITH PPCM**

| **ECG feature** | **All Women with PPCM**  **(% of total ECGs, n=68)** |  | **Women with Abnormal ECGs**  **(% of abnormal ECGs, n=56)** | |
| --- | --- | --- | --- | --- |
| **Normal sinus rhythm** | 26 (38.2) |  | 13 (23.2) | |
| **Sinus tachycardia** | 37 (54.4) |  | 37 (66.1) | |
| **Sinus bradycardia** | 3 (4.4) |  | 3 (5.4) | |
| **Sinus arrhythmia** | 2 (2.9) |  | 2 (3.6) | |
| **Mean heart rate (bpm)** | 103 |  | 108 | |
| **Atrial fibrillation** | 0 |  | 0 | |
| **Other arrhythmia** | 11 (16.2) |  | 11 (19.6) | |
| **Normal axis** | 57 (83.8) |  | 46 (82.1) | |
| **Abnormal axis**  ***Left axis deviation***  ***Right axis deviation*** | 10 (14.7)  *2*  *8* |  | 10 (17.9)  *2*  *8* | |
| **Left atrial enlargement** | 9 (13.2) |  | 9 (16.1) | |
| **Right atrial enlargement** | 2 (2.9) |  | 2 (3.6) | |
|  |  |  |  | |
| **Left ventricular hypertrophy** | 5 (7.4) | | | 5 (8.9) |
| **Long PR** | 1 (1.5) | | | 1 (1.8) |
| **Long QTc**  ***Average QTc*** | 27 (39.7)  *451* | | | 27 (48.2)  *455* |
| **Left bundle branch block** | 1 (1.5) | | | 1 (1.8) |
| **Right bundle branch block** | 0 | | | 0 |
| **Pathologic Q waves** | 9 (13.2) | | | 9 (16.1) |
| **T wave inversion** | 17 (25.0) | | | 17 (30.4) |
| **Poor R wave progression** | 16 (23.5) | | | 16 (28.6) |
| **Positive T wave in aVR**  ***T wave amplitude if +***  ***(average in mm)*** | 10 (14.7)  *0.8* | | | 10 (17.9)  *0.8* |
